# Overlap of high-risk individuals predicted by family history, and genetic and non-genetic breast cancer risk prediction models: implications for risk stratification

**DOI:** 10.1186/s12916-022-02334-z

**Published:** 2022-04-26

**Authors:** Peh Joo Ho, Weang Kee Ho, Alexis J. Khng, Yen Shing Yeoh, Benita Kiat-Tee Tan, Ern Yu Tan, Geok Hoon Lim, Su-Ming Tan, Veronique Kiak Mien Tan, Cheng-Har Yip, Nur-Aishah Mohd-Taib, Fuh Yong Wong, Elaine Hsuen Lim, Joanne Ngeow, Wen Yee Chay, Lester Chee Hao Leong, Wei Sean Yong, Chin Mui Seah, Siau Wei Tang, Celene Wei Qi Ng, Zhiyan Yan, Jung Ah Lee, Kartini Rahmat, Tania Islam, Tiara Hassan, Mei-Chee Tai, Chiea Chuen Khor, Jian-Min Yuan, Woon-Puay Koh, Xueling Sim, Alison M. Dunning, Manjeet K. Bolla, Antonis C. Antoniou, Soo-Hwang Teo, Jingmei Li, Mikael Hartman

**Affiliations:** 1 Genome Institute of Singapore, Human Genetics, Singapore, Singapore; 2 Saw Swee Hock School of Public Health, National University of Singapore and National University Health System, Singapore, Singapore; 3 Department of Surgery, Yong Loo Lin School of Medicine, National University of Singapore and National University Health System, Singapore, Singapore; 4 Cancer Research Malaysia, 1 Jalan SS12/1A, 47500 Subang Jaya, Selangor Malaysia; 5 School of Mathematical Sciences, Faculty of Science and Engineering, University of Nottingham Malaysia, Jalan Broga, 43500 Semenyih, Selangor Malaysia; 6 Department of General Surgery, Sengkang General Hospital, Singapore, Singapore; 7 Department of Breast Surgery, Singapore General Hospital, Singapore, Singapore; 8 Division of Surgery and Surgical Oncology, National Cancer Centre Singapore, Singapore, Singapore; 9 Department of General Surgery, Tan Tock Seng Hospital, Singapore, 308433 Singapore; 10 Lee Kong Chian School of Medicine, Nanyang Technology University, Singapore, Singapore; 11 Institute of Molecular and Cell Biology, Singapore, Singapore; 12 KK Breast Department, KK Women’s and Children’s Hospital, Singapore, 229899 Singapore; 13 Division of Breast Surgery, Changi General Hospital, Singapore, Singapore; 14 Subang Jaya Medical Centre, Subang Jaya, Selangor Malaysia; 15 Department of Surgery, Faculty of Medicine, University of Malaya, Kuala Lumpur, Malaysia; 16 Universiti Malaya Cancer Research Institute, Kuala Lumpur, Malaysia; 17 Division of Radiation Oncology, National Cancer Centre Singapore, Singapore, Singapore; 18 Division of Medical Oncology, National Cancer Centre Singapore, Singapore, Singapore; 19 Cancer Genetics Service, National Cancer Centre Singapore, Singapore, Singapore; 20 Department of Diagnostic Radiology, Singapore General Hospital, Singapore, Singapore; 21 Department of Surgery, University Surgical Cluster, National University Hospital, Singapore, Singapore; 22 Healthy Longevity Translational Research Programme, Yong Loo Lin School of Medicine, National University of Singapore, Singapore, Singapore; 23 UPMC Hillman Cancer Center, Pittsburgh, PA USA; 24 Department of Epidemiology, University of Pittsburgh Graduate School of Public Health, Pittsburgh, PA USA; 25 Singapore Institute for Clinical Sciences, Agency for Science Technology and Research (A*STAR), Singapore, 117609 Singapore; 26 Centre for Cancer Genetic Epidemiology, Department of Oncology, University of Cambridge, Cambridge, UK; 27 Centre for Cancer Genetic Epidemiology, Department of Public Health and Primary Care, University of Cambridge, Cambridge, UK; 28 Department of Surgery, Faculty of Medicine, University of Malaya, Jalan Universiti, 50630 Kuala Lumpur, Malaysia

**Keywords:** Breast cancer, Polygenic risk score, Gail model, Protein-truncating variants, Risk-based screening

## Abstract

**Background:**

Family history, and genetic and non-genetic risk factors can stratify women according to their individual risk of developing breast cancer. The extent of overlap between these risk predictors is not clear.

**Methods:**

In this case-only analysis involving 7600 Asian breast cancer patients diagnosed between age 30 and 75 years, we examined identification of high-risk patients based on positive family history, the Gail model 5-year absolute risk [5yAR] above 1.3%, breast cancer predisposition genes (protein-truncating variants [PTV] in *ATM*, *BRCA1*, *BRCA2*, *CHEK2*, *PALB2*, *BARD1*, *RAD51C*, *RAD51D*, or *TP53*), and polygenic risk score (PRS) 5yAR above 1.3%.

**Results:**

Correlation between 5yAR (at age of diagnosis) predicted by PRS and the Gail model was low (*r*=0.27). Fifty-three percent of breast cancer patients (*n*=4041) were considered high risk by one or more classification criteria. Positive family history, PTV carriership, PRS, or the Gail model identified 1247 (16%), 385 (5%), 2774 (36%), and 1592 (21%) patients who were considered at high risk, respectively. In a subset of 3227 women aged below 50 years, the four models studied identified 470 (15%), 213 (7%), 769 (24%), and 325 (10%) unique patients who were considered at high risk, respectively. For younger women, PRS and PTVs together identified 745 (59% of 1276) high-risk individuals who were not identified by the Gail model or family history.

**Conclusions:**

Family history and genetic and non-genetic risk stratification tools have the potential to complement one another to identify women at high risk.

**Supplementary Information:**

The online version contains supplementary material available at 10.1186/s12916-022-02334-z.

## Background

Multiple randomized controlled trials have shown that screening mammography reduces mortality from breast cancer for women who are over 50 years old [1]. Screening programs have been set up in different countries since the 1970s with recommendations on the screening interval and the age to start screening [2]. There is a strong consensus that women above 50 should attend routine screening. Recommendations for this group of women have remained largely unchanged for over 40 years [1, 2].

The benefit of screening mammography for younger women aged 40 to 49 years is less clear [1]. Recommendations for screening mammography across different countries and time periods are inconsistent and subject to change [2, 3]. In many cases, a woman is to make a personal choice based on risk factors such as personal and family history of the disease, often with the help of professional advice from a doctor [1].

With advances made in disease prediction, the approach to breast screening is now leaning towards a tailored, individual risk-based approach. For example, mammographic breast density, which refers to the proportion of fibroglandular breast tissue compared to fat seen on mammography, is a risk factor for breast cancer and is increasingly communicated to screening participants [4, 5]. Women with dense breasts are informed of their increased risk of breast cancer development and reduced sensitivity of mammography to detect breast cancer so that they can make a better-informed decision as to whether they should undergo supplemental imaging screening adjunct to mammography [5].

The risk of breast cancer is multifactorial. Apart from mammographic density, other known conventional risk factors include family history, menarche age, menopause age, height, body mass index, age at first childbirth, menopausal hormone therapy, and benign breast disease [6]. Many of these factors have been incorporated into prediction models to estimate the personal risk of developing breast cancer [7].

Breast cancer has a significant genetic component. It has been estimated that 27–31% of breast cancer risk may be explained by heritable factors [8, 9]. Frequently described breast cancer predisposition genes that are highly penetrant include *ATM*, *BRCA1*, *BRCA2*, *CHEK2*, *PALB2*, *BARD1*, *RAD51C*, *RAD51D*, and *TP53* [10]. However, pathogenic mutations in these genes are rare in the population. Polygenic risk scores (PRS) computed from another class of genetic variants that are of smaller effect sizes individually but more common in the population have shown promise to add information to better stratify individuals with different breast cancer risks as compared to age-based screening programs [11–13].

Non-genetic risk prediction models are attractive as they are non-invasive and are easier to implement in a general population or primary care screening setting. Currently, breast cancer risk prediction is predominantly based on information on age, family history, lifestyle, and reproductive factors. These data can be collected at a low cost. There is evidence that genetics contribute to risk prediction but the data generation will incur additional costs to individuals or the health system. Hence, there is a need to evaluate how much information genetics can add to the identification of high-risk individuals over non-genetic risk factors. In this case-only analysis involving 7600 Asian breast cancer patients, we look at the overlap of individuals with a family history of breast cancer and those identified to be at high risk based on family history, the Gail model, breast cancer predisposition genes, and polygenic risk score.

## Methods

### Study populations

Breast cancer patients from two multi-ethnic populations recruited in Singapore and Malaysia, with ethnic groups of Chinese, Malay, and Indian descent were included in this study. These patients were recruited as part of the Singapore Breast Cancer Cohort (SGBCC) [14] and the Malaysian Breast Cancer Genetic Study (MyBrCa) [15]. Controls were recruited from the Singapore Multi-Ethnic Cohort (MEC) study [16] and the Malaysian Mammography Study (MyMammo) [15] for the calculation of the mean PRS and SD for the standard population. A prospective cohort study of healthy individuals—the Singapore Chinese Health Study (SCHS)—was used as a validation cohort [17]. Full details on each study and their respective DNA isolation and genotyping protocols are available in Additional file 1 [14–23].

### Carriership of protein-truncating variants in nine breast cancer predisposition genes

Target-enriched sequencing libraries of germline DNA for the breast cancer cases (SGBCC and MyBrCa) were prepared at the Centre for Cancer Genetic Epidemiology (University of Cambridge) as part of a larger effort (Breast Cancer Risk after Diagnostic Gene Sequencing) [10]. Details of the library preparation, sequencing, variant calling, and quality control methods are given in Dorling et al. [10]. Protein-truncating variants (PTVs) include nonsense single-nucleotide variants (SNVs), frameshift insertions or deletions (indels), and splice-disrupting SNVs. PTVs occurring in the last exon of each gene were excluded to avoid including variants that do not lead to nonsense-mediated decay. Here, we studied nine genes found to be relevant for breast cancer risk as reported in Dorling et al. [10]—*ATM*, *BRCA1*, *BRCA2*, *CHEK2*, *PALB2*, *BARD1*, *RAD51C*, *RAD51D*, and *TP53*—in 192 and 193 breast cancer patients from SGBCC and MyBrCa, respectively (Additional file 2 – Tables S1 and S2) [10].

### Polygenic risk score (PRS)

PRS is estimated as the weighted sum of effect alleles in 313 single nucleotide polymorphisms (SNPs) found to be associated with breast cancer, using plink (version 3) with the *scoresum* option (full details in Additional file 1) [19].

### Gail model

Data on breast cancer risk factors (age at menarche, age at first live birth, ever had a biopsy, and family history of breast cancer) were obtained from structured questionnaires. Family history of breast cancer was available as a binary variable (yes/no). Weights (logistic regression coefficients derived from the Gail model) and attributable risks of Asian-Americans (“Asian.AABCS”, *BCRA* package in R) were used in the calculation of the Gail model absolute risk [22].

### Five-year absolute risk

Five-year absolute risk at the age of breast cancer diagnosis, for both PRS and the Gail model relative risk, was estimated for breast cancer patients aged between 30 and 75 from SGBCC and MyBrCa. The absolute risk was based on ethnic-specific or overall breast cancer incidence rates (period of 2013 to 2017) for Singapore Citizens and mortality rates (the year 2016) in Singapore (Additional file 3) [20, 23]. Both incidence and mortality rates were recorded in 5-year intervals. The 5-year absolute risk based on PRS was estimated using an iterative method detailed by Mavaddat et al. [13]. The 5-year absolute risk predicted by the Gail model was estimated using the method in the *BCRA* package in R [22]. Details on the calculation of 5-year absolute risks are available in Additional file 1.

### Statistical analysis

#### Individual risk of developing breast cancer over 5 years based on PRS and Gail model

A comparison between the 5-year absolute risks predicted by PRS and the Gail model was examined using the Wilcoxon signed-rank test. Spearman’s correlation coefficient was estimated.

#### Classification of breast cancer patients into high- or low-risk groups

To illustrate a potential screening program where only high-risk individuals are screened, individuals were classified into high or low breast cancer risk groups. The following criteria were used to define high-risk groups: (1) at least one first degree relative diagnosed with breast cancer or ovarian cancer (effect of family history), (2) 5-year absolute risk above 1.3% estimated by PRS (effect of common genetic variants), (3) 5-year absolute risk above 1.3% estimated by Gail risk model (effect of non-genetic variants), and (4) carriership of PTV in *ATM*, *BRCA1*, *BRCA2*, *CHEK2*, *PALB2*, *BARD1*, *RAD51C*, *RAD51D*, or *TP53* (effect of rare genetic variants). The current recommendation is for women aged between 40 and 49 years to start screening when their individual 5-year risk is the same as or exceeds that of an average 50-year-old woman [24]. The threshold of 1.3% is equivalent to the 5-year absolute risk of developing breast cancer for an average Caucasian woman aged 50 years [24]. Cohen’s kappa was used to test pairwise concordance between the classification of breast cancer patients based on PRS and the Gail model [25].

#### Agreement between criteria

The agreement between pairs of different criteria to identify breast cancer patients at high risk was estimated using kappa scores. The incremental proportions of breast cancer patients identified as being at high risk are presented for 5-year age intervals.

Analyses were performed on a combined dataset of SGBCC and MyBrCa breast cancer patients and repeated for each cohort separately. In addition, the analyses were repeated for the prospective SCHS study, but without PTV.

Analysis was performed in R version 4.0.3.

## Results

### Description of SGBCC and MyBrCa breast cancer patients diagnosed between ages 30 and 75

Table 1 describes the summary characteristics of the combined 7600 breast cancer patients included in the case-only analysis from SGBCC (*n*=4284) and MyBrCa (*n*=3316). The median age at diagnosis was 53 years (interquartile range [IQR]: 45 to 59). Fifteen percent of the patients had first-degree relatives with breast (*n*=1133) and 2% with ovarian cancer (*n*=151). Five percent (*n*=385) of our breast cancer patients were PTV carriers with one or more of the nine known breast cancer predisposition genes [10] (Additional file 2 – Table S3). Additional file 2 – Table S4 describes the attributes of the breast cancer patients of three participating studies separately.

**Table 1:** Description of 7600 breast cancer patients diagnosed between ages 30 and 70. Patients were recruited as part of the Singapore Breast Cancer Cohort (SGBCC) and the Malaysian Breast Cancer Genetic Study (MyBrCa). The description of the study is in Additional file Table S4. *IQR* interquartile range

Variable	Statistic
Median age at diagnosis (IQR)	52 (45–59)
**Study**	
SGBCC	4284 (56%)
MyBrCa	3316 (44%)
**Case-type**	
Incidence (enrolled within one year of diagnosis)	4511 (59%)
Prevalence	3087 (41%)
Missing	2 (0%)
**Ethnicity**	
Chinese	5724 (75%)
Malay	1145 (15%)
Indian	645 (8%)
Other	82 (1%)
Unknown	4 (0%)
**Age at menarche, years,** ***n*** **(%)**	
≥14	2212 (29%)
12 to 13	3968 (52%)
<12	803 (11%)
Unknown	617 (8%)
**Age at first birth, years,** ***n*** **(%)**	
<20	335 (4%)
20 to 25	1479 (19%)
25 to 30	2289 (30%)
≥30	1792 (24%)
Nulliparous	1294 (17%)
Unknown	411 (5%)
**Family history of breast cancer**	
No	6364 (84%)
Yes	1132 (15%)
**Number of first degree relatives with ovarian cancer**	
No	7444 (98%)
Yes	151 (2%)
**Carriers of breast cancer predisposition genes,** ***n*** **(%)**	
Non-carrier	7215 (95%)
Carrier	385 (5%)
**Median 5-year absolute risk (IQR)**	
Gail model relative risk	0.9 (0.7–1.2)
Polygenic risk score (PRS)	1.1 (0.7–1.6)

### Low correlation between breast cancer absolute risk by PRS and the Gail model by study

**Fig. 1:**
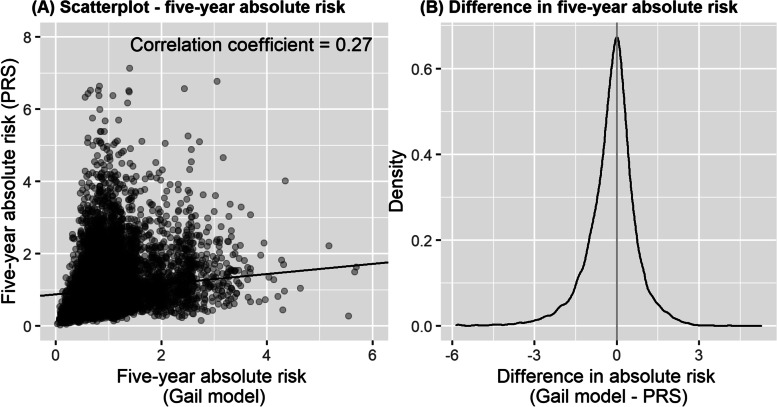
Comparing the 5-year absolute risk prediction using the Gail model and polygenic risk score (PRS). **A** A scatterplot of the 5-year absolute risk of the Gail model against PRS, by cohorts (the Singapore Breast Cancer Cohort [SGBCC] and the Malaysian Breast Cancer Genetic Study [MyBrCa]). The linear fitted lines (solid: SGBCC, dashed: MyBrCa) and Spearman’s correlation coefficients by cohort are shown. **B** The difference between the 5-year absolute risk of the Gail model and the polygenic risk score

The median 5-year absolute risk was 1.1 (IQR: 0.7 to 1.6) by PRS and 0.9 (IQR: 0.7 to 1.2) by the Gail model (Table 1). Spearman’s correlation coefficient between the 5-year absolute risks by PRS and the Gail model was low (*r*=0.27) (Fig. 1A). The median difference between the 5-year absolute risks estimated by PRS and the Gail model was − 0.097 (IQR: − 0.591 to 0.296, Wilcoxon signed-rank test *p* value <0.001) (Fig. 1B). Comparison by cohorts (SGBCC and MyBrCa) are shown in Additional file 2 – Fig. S1.

### PRS and the Gail model identified larger proportions of unique high-risk individuals among breast cancer patients compared to PTV carriership and family history

**Fig. 2:**
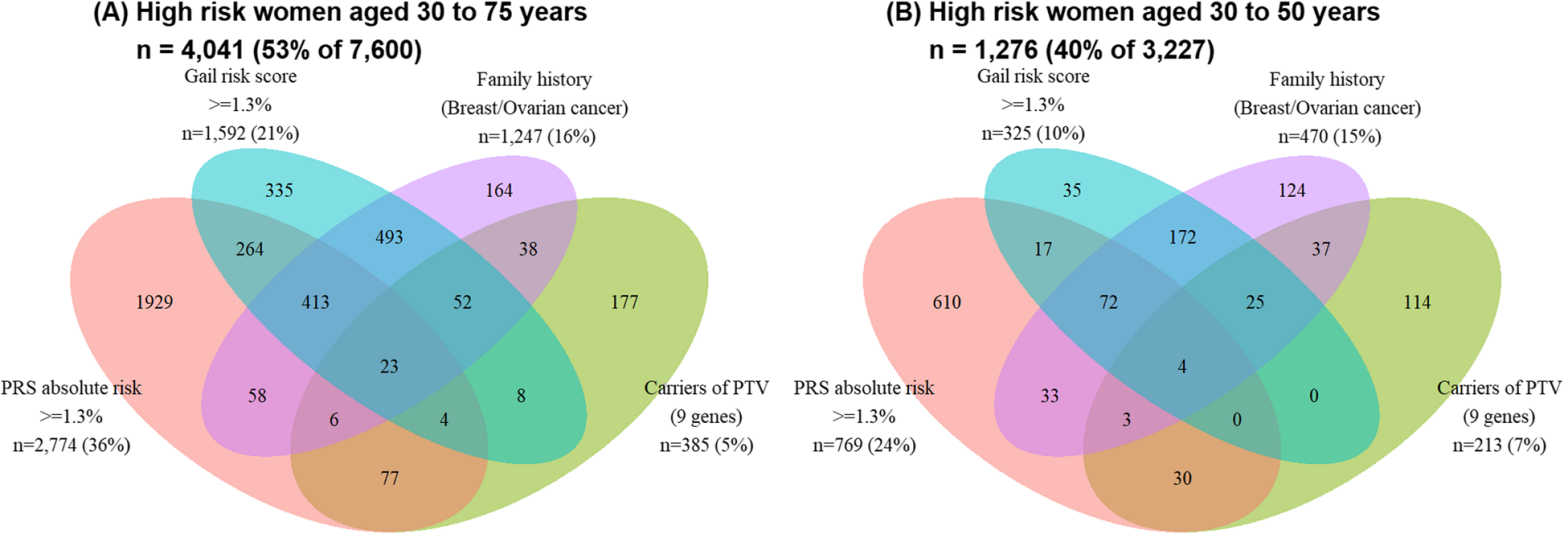
Venn diagram of breast cancer patients at high risk of breast cancer. Patients were identified as being at high risk by first-degree family history of breast cancer, protein-truncating variant (PTV) carriership in nine breast cancer predisposition genes (*ATM*, *BRCA1*, *BRCA2*, *CHEK2*, *PALB2*, *BARD1*, *RAD51C*, *RAD51D*, and *TP53*), and 5-year absolute risk by polygenic risk score (PRS) or Gail risk score. **A** High-risk breast cancer patients. **B** A subset of high-risk young breast cancer patients

Approximately half of all (53%, *n*=4041) breast cancer patients were considered high risk by any of the four risk classification criteria studied (Fig. 2A). PRS (5-year absolute risk ≥1.3%) alone identified the largest proportion of high-risk patients (*n*=2774 (36%)) (Fig. 2A). This was followed by Gail model (5-year absolute risk ≥1.3%, *n*=1592 (21%)), positive family history (*n*=1247 (16%)), and PTV carriership (*n*=385 (5%)) (Fig. 2A). Among 385 PTV carriers, 110 (28%) were considered high risk by PRS. We observed poor or slight concordance between each pair of criteria in classifying patients as high risk; Cohen’s kappa ranged from − 0.025 to 0.095 with the exception of 0.621 for the Gail model with a family history of breast or ovarian cancer (Additional file 2 – Table S5).

### Family history and genetic risk stratification models identified much younger breast cancer patients at high risk

For women below standard mammography screening entry age of 50 years (age 30 to 50 years; *n*=3227), the breast cancer risk stratification tools studied identified 40% (*n*=1276) of the breast cancer patients to be high risk. Risk stratification by positive family history, PTV carriership, and 5-year absolute risk ≥1.3% by PRS or the Gail model identified 470 (15%), 213 (7%), 769 (24%), and 325 (10%) unique breast cancer patients who were considered at high risk of breast cancer, respectively (Fig. 2B). The genetic risk stratification models, PTV carriership and PRS, identified 114 and 610 additional high-risk individuals that were not identified by family history and the Gail model. Thirty-seven individuals were considered high risk based on both PTV carriership and PRS. Slight concordance was observed between the criteria based on the 5-year absolute risks by PRS and the Gail model in classifying young patients as high risk (Cohen’s kappa: 0.052, *p*<0.001, Additional file 2 – Table S6). Additional file 2 – Fig. S2 presents the classification of high-risk patients by study.

### Proportion of breast cancer patients identified as being at high risk within 5-year age groups

**Fig. 3:**
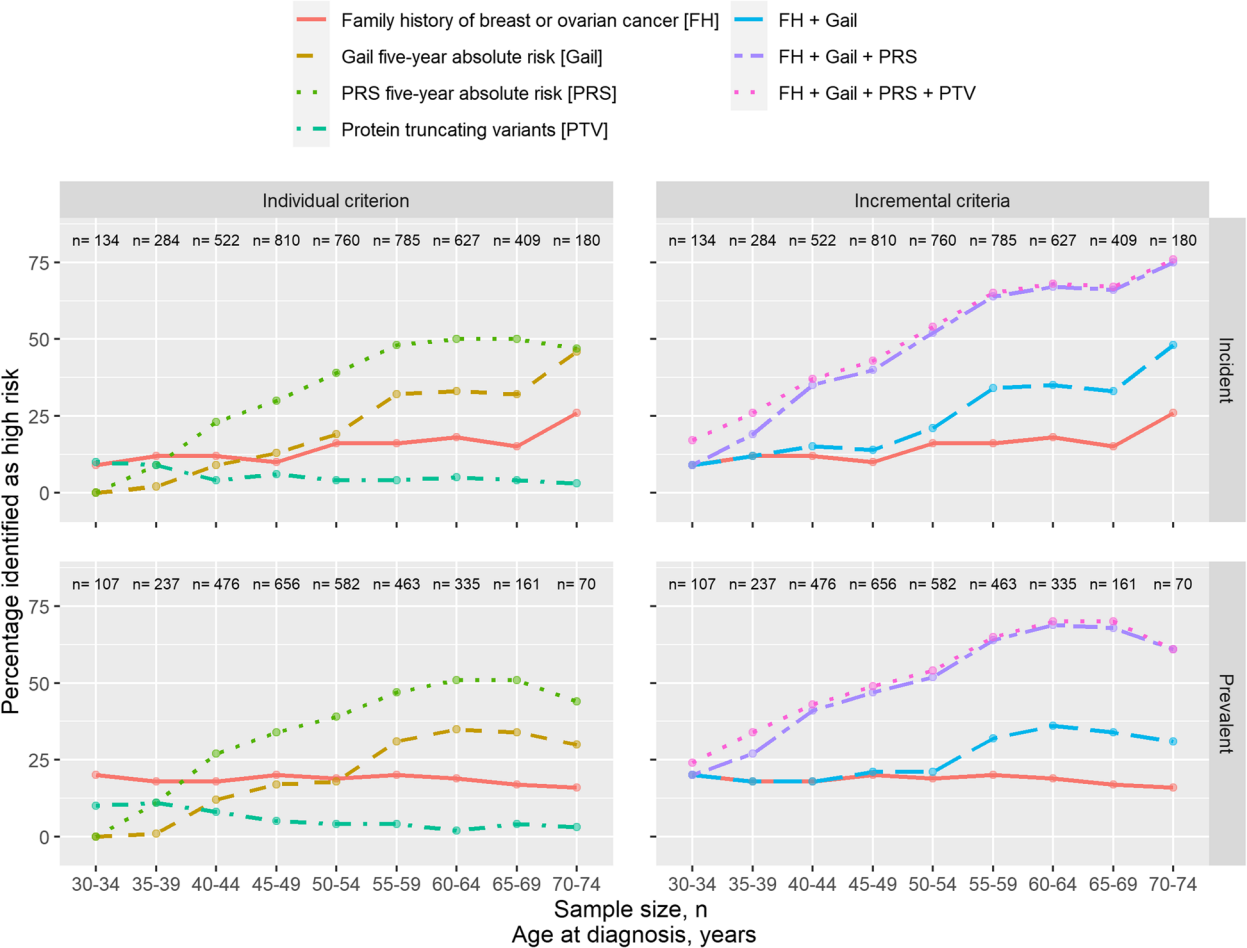
Proportion of breast cancer patients identified as being at high-risk within 5-year age groups. Proportions are presented by case-type (incident [i.e. enrolled within one year of diagnosis date] and prevalent). Criteria: (1) at least one first degree relative diagnosed with breast or ovarian cancer [FH], (2) 5-year absolute risk above 1.3% estimated by PRS [PRS], (3) 5-year absolute risk above 1.3% estimated by Gail risk model [Gail], and (4) carriership of PTV in *ATM*, *BRCA1*, *BRCA2*, *CHEK2*, *PALB2*, *BARD1*, *RAD51C*, *RAD51D*, or *TP53* [PTV]

As an individual criterion, the 5-year absolute risk, by PRS or the Gail model, identified the largest proportion of high-risk patients in the breast cancer screening age group (50 to 65 years) (Fig. 3). The 5-year absolute risk by PRS identified the largest proportion of high-risk patients in age groups including ages of 40 years and above, with the highest proportion (50%) in the age group 60 to 64 years (Fig. 3). For both criteria (PRS or the Gail model), the proportion identified as at high risk decreases in the younger age groups, with less than 10% being identified in the youngest age group 30 to 34 years. Family history remained at the level of less than 20% for all age groups younger than 70 years.

More than 50% in the older age groups (50 years and above) of breast cancer were identified as at high risk when we added information of each criterion sequentially (starting with family history → above 1.3% 5-year absolute risk by the Gail model → above 1.3% 5-year absolute risk by PRS → PTV carriership) (Fig. 3). The addition of the PRS criterion produced the largest change in proportion identified as at high risk (Fig. 3). Additional file 2 – Figs. S3 and S4 show the proportion of high-risk patients in each age group by study.

### Validation in SCHS, a prospective cohort of healthy individuals

Summary characteristics and allele frequencies corresponding to the 313 variants included in the PRS of the 10,213 women from SCHS are presented in Additional file 2 – Tables S4 and S7, respectively. Four percent (*n*=418) developed breast cancer over a median follow-up of 20 years (IQR: 18 to 21), of which 19% (*n*=81) occurred within 5 years of recruitment. Among the 81, 38% (*n*=31) were above 1.3% 5-year absolute risk by PRS, while only 6% were above 1.3% 5-year absolute risk by the Gail model, suggesting a higher performance of the PRS than the Gail model for breast cancer risk stratification for middle-aged or older Chinese women.

Due to the small number of events within 5 years of recruitment, we studied all breast cancer patients ignoring time to event. The proportion of patients with above 1.3% 5-year absolute risk by PRS (in SCHS’s breast cancer patients) was higher than that observed in SGBCC and MyBrCa’s patients (SCHS = 41%, SGBCC + MyBrCa = 36%) (Additional file 2 – Fig. S5). However, the proportion of patients with above 1.3% 5-year absolute risk by the Gail model was lower (SCHS = 7%, SGBCC + MyBrCa = 21%).

**Fig. 4:**
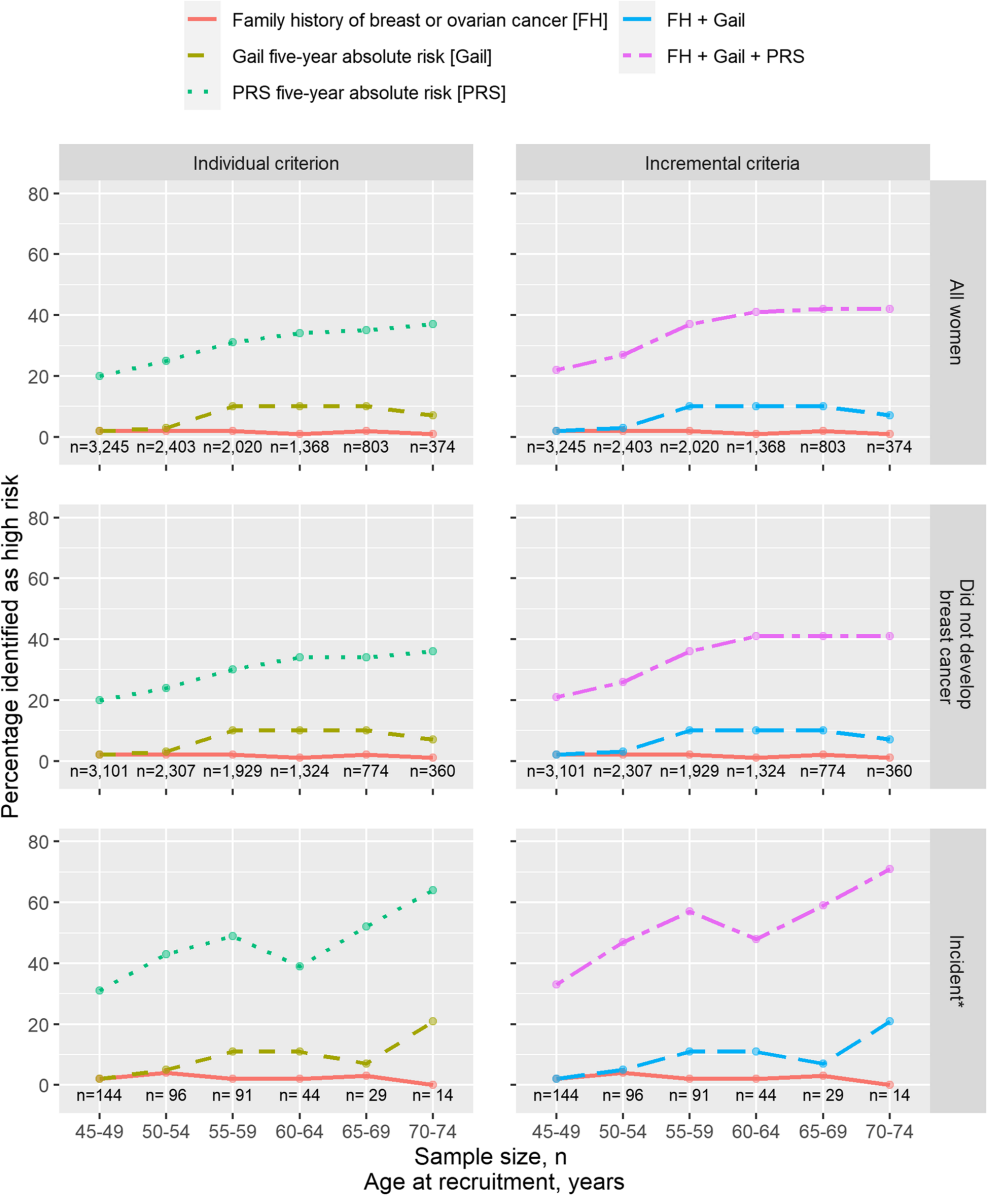
Proportion of breast cancer patients from the validation dataset identified as being at high risk. The prospective cohort of healthy women—the Singapore Chinese Health Study [SCHS]—was used for validation. Criteria for high-risk: (1) at least one first degree relative diagnosed with breast or ovarian cancer [FH], (2) 5-year absolute risk above 1.3% estimated by the polygenic risk score [PRS], and (3) 5-year absolute risk above 1.3% estimated by Gail risk model [Gail]. PRS is standardized with mean and standard deviation of Chinese controls from the Singapore and Malaysia dataset. *Note: This plot uses age at recruitment; breast cancer may not occur within 5 years of recruitment

Identifying women (with no personal history of breast cancer, *n*=10,213) at above 1.3% 5-year absolute risk by PRS resulted in a larger proportion of high-risk women (*n*=2761, 27%) as compared to using the Gail model (6%) (Additional file 2 – Fig. S5). However, 6% (*n*=172, of 2761) of these high-risk women identified by PRS developed breast cancer, which was similar to the 5% (*n*=28, of 587) by the Gail model, and 6% (*n*=11, of 180) by family history. Adding PRS to risk stratification identified 165 more breast cancer cases. Figure 4 shows the percentage of women identified as high risk by adding the Gail model and PRS to family history across different age groups. The Gail model identified up to 10% more high-risk individuals, while PRS identified an additional ~27% on top of the Gail model and family history.

## Discussion

Currently, in many countries, population-based mammography screening is recommended based on age alone. However, not every woman is at the same level of risk of developing breast cancer. In practice, family history of the disease is widely used as a risk assessment tool. Breast cancer risk of women with a sister or a mother with breast cancer is reported to be approximately twice as high as those who do not have first-degree family members diagnosed with the disease [26]. In addition, family history information of high quality is reported to be highly correlated to the carriership of actionable genomic variants [21]. Prediction models using breast cancer risk factor information collected using questionnaires, such as the Gail model, are also widely used [27]. On the individual level, these risk estimates are encouraged to be included in conversations with clinicians to help make informed decisions about potential interventions, including chemoprevention with tamoxifen [27, 28].

While family history and conventional breast cancer risk factors may change over time and thus require updates and reassessments, an individual’s genetic risk based on either established breast cancer predisposition genes or PRS may be determined at birth. However, the implementation of genetic tests in population-wide screening is highly debatable. Pathogenic variants in high-penetrance breast cancer genes are rare; hence, most women in the general population will not benefit and may develop a false sense of security [29]. Previously, the evidence that common genetic variants (used in the calculation of PRS) provide superior risk stratification over conventional breast cancer risk factors is lacking [30, 31]. There was also no consensus on which variants to include in the PRS calculation. However, recent international mega-consortia studies examining over a hundred thousand women show that the tail ends of PRS enable more precise risk differentiation [11–13].

With the latest developments in genetic risk prediction, it is timely to consider whether every woman in the general population should be genetically screened for high-risk genes and the use of PRS in a screening program. Our findings show that both genetic and conventional risk stratification tools have their own merits and are able to identify unique individuals at risk. Each risk assessment tool is a partial predictor at best. The inclusion of multiple predictive tools can pick up additional high-risk individuals who are missed out from using any one tool alone. In our study, family history and genetic risk perform better for women below age 50, as compared to the Gail model. This is noteworthy as the entry age for subsidized breast screening in many countries is 50 years. Genetic risk profiles will help younger women in making informed decisions on whether they should start screening at an earlier age. High-risk individuals may benefit from specific recommendations or interventions based on their personal breast cancer risk profiles.

In countries where breast screening uptake is low, breast cancer risk assessment tools function more than just predictive scores. The knowledge of breast cancer risk on an individual level may serve as a tool to motivate behavioral change. For example, a Finnish study studied the impact of genetic and non-genetic personal risk scores for cardiovascular diseases on health behavior in over 7000 participants. The results show that risk-reducing behavior is observed in participants across all risk strata, although more individuals at high risk made a health behavioral change (42.6% vs 33.5% of individuals not at high risk) [32]. The contributions of genetic and non-genetic risk profile feedback were reported to be independent of each other [32], further supporting the inclusion of both genetic and non-genetic risk factors for stratification in screening programs.

In terms of mammography screening, PRS has been reported to perform well at identifying the women who are most likely to benefit from this mode of detection [33]. The association between PRS and tumor characteristics in our study confirms this observation. Not all tumors grow at the same rate. Despite the advances in technology, routine mammography screening on average fails to detect ~10–30% of all breast cancers [5, 34]. Some of these missed tumors are interval cancers that are diagnosed between two screening episodes [35]. Women at high risk based on PRS will thus benefit from increased screening frequency and compliance to screening.

The main strength of our study is that this is one of the largest and most well-characterized breast cancer cohorts of Asian women. However, many of the breast cancer risk assessment tools and the PRS are established based on European populations and their utility in Asian breast cancer populations remains unclear. Approximately half of the breast cancer population were identified as high risk, suggesting that other factors not considered in the risk prediction models (e.g., mammographic density, physical activity, alcohol, smoking) studied may be responsible. We classified breast cancer patients into risk categories based on 5-year absolute risks at the age of breast cancer diagnosis; this may not be representative of women without breast cancer. While we are likely to overestimate the 5-year absolute risk, results from the prospective cohort (SCHS) support the use of genetic factors on top of family history and the Gail model. As this is a case-only cohort, the proportions from the risk classification analysis are not representative of the general population, where most women will be classified as low risk. Nonetheless, this will not affect the comparison of how different criteria identify women at high risk.

While this study’s main focus was to highlight the lack of an overlap between high-risk women identified by genetic and non-genetic risk factors, it is worth noting that other works in the field have studied the potential improvements in risk prediction by combining different risk factors. For instance, Choudhury et al. explored the value of adding mammographic density and PRS to classical risk factors in a population of women of European ancestry [36]. In a more recent study, Yang et al. assessed the performance of breast cancer risk prediction models incorporating genetic and non-genetic risk factors in 20,444 breast cancer cases and 106,450 controls from the Asia Breast Cancer Consortium [37]. These developments are complementary to the findings of this study and will help pave the way for more patient-centric, data-driven healthcare systems in the future.

## Conclusions

In summary, our assessment shows that family history and genetic and non-genetic risk stratification tools have the potential to complement one another to identify women at high risk in breast screening programs. The results add to the growing body of evidence to support a paradigm shift from an approach that is age-based to risk-based.

## Supplementary Information

**Additional file 1.** Details on the study populations, DNA isolation and genotyping, polygenic risk score (PRS) calculation, and methods to obtain absolute risk by PRS and the Gail model.

**Additional file 2: Table S1.** Targeted regions of gene panel (hg19). **Table S2.** List of predicted protein-truncating variants. **Table S3.** Carriers of protein truncating variants. **Table S4. **Characteristics of cohorts. **Tables S5** and **S6.** Agreement between the five criteria in identifying breast cancer patients at high risk of breast cancer. **Table S7.** List of 313 variants in the polygenic risk score. **Figure S1**. Comparing the five-year absolute risk prediction. **Figures S2** and **S5.** Venn diagram of breast cancer patients at high risk of breast cancer. **Figures S3** and **S4.** Proportion of breast cancer patients identified as being at high risk.

**Additional file 3.** The incidence and mortality rates of Singapore.

**Additional file 4: Table SM1** and **SM2.** List of 313 variants in the polygenic risk score. **Table SM3.** Mean and standard deviation of polygenic risk scores. **Table SM4.** Relative risk of the percentile corresponding to Figure SM4. **Figure SM1**-**SM3.** Flowcharts for individuals selected. **Figure SM4** and **SM5.** Five-year absolute risk of developing breast cancer.

## Data Availability

The data that support the findings of this study are available on request from Mikael Hartman (ephbamh@nus.edu.sg). The data are not publicly available due to privacy or ethical restrictions.
