# Supplementary material for: Overlap of high-risk individuals predicted by family history, and genetic and non-genetic breast cancer risk prediction models: implications for risk stratification
Source: BMC Med. 2022 Apr 26;20:150. doi: 10.1186/s12916-022-02334-z (PMC9040206; doi:10.1186/s12916-022-02334-z)
Supplement: Supplementary file 1 — Additional file 1. Details on the study populations, DNA isolation and genotyping, polygenic risk score (PRS) calculation, and methods to obtain absolute risk by PRS and the Gail model. [file 12916_2022_2334_MOESM1_ESM.pdf]

Supplementary Methods

Contents

Supplementary Methods 1
- Study populations 2
- DNA isolation and genotyping 3
- Polygenic risk score (PRS) 4
- Five-year absolute risk 4
- Edited codes of BCRA package from R 5

Study populations

Singapore Breast Cancer Cohort (SGBCC)

Breast cancer patients from Singapore were from the SGBCC study, where 7,768 female breast cancer patients were recruited between 2010 and 2016 from six restructured hospitals: National University Hospital, KK Women's and Children's Hospital, Tan Tock Seng hospital, Singapore General Hospital, National Cancer Centre Singapore, Changi General Hospital (cohort described in [14]. Seventy-six percent of all breast cancer cases in Singapore are treated in these hospitals [14]. A subset of 5,931 women (76.3%) consented to provide blood or saliva. We excluded 1,441 individuals who were not genotyped (biospecimens not available for retrieval from repository, or biospecimens were not collected before genotyping or sequencing experiments), 21 who failed genotyping quality control, 10 who were genetic duplicates, 5 who were duplicated due to recruitment at multiple hospitals, and 5 who withdrew consent. Of the 4,449 women remaining, 4,284 were diagnosed between the ages of 30 and 75 years (**Additional file 4** – **Figure SM1A**). Forty-three percent (n=1,852) of the breast cancer cases were diagnosed before study initiation.

Singapore Multi-Ethnic Cohort (MEC) study

To obtain means and standard deviations of PRS of the general population, population-based non-breast cancer controls from the MEC study was used. The MEC study is a population-based cohort of the general adult population of Singapore, with participants aged between 21 and 75 at enrollment (cohort described in [16]. Women enrolled in MEC were ethnicity and age (+/- 5 years) matched controls to SGBCC breast cancer cases [16]. Among 4,099 women were never diagnosed with breast cancer, 4,098 had genetic information (**Additional file 4** – **Figure SM1B**).

Malaysian Breast Cancer Genetic Study (MyBrCa)

MyBrCa consisted of 3,783 breast cancer patients, recruited between 2002 and 2016. Details are described by Tan *et al*. [15]. Patients were recruited from two participating hospitals in Selangor, Malaysia (University Malaya Medical Centre [UMMC], a public hospital, and Subang Jaya Medical Centre [SJMC],

a private hospital). Patients without available genotyping data were excluded (n=271). A subset of 3,316 patients were aged between 30 and 75 years at diagnosis (**Additional file 4** – **Figure SM2A**).

Malaysian Mammography Study (MyMammo)

MyMammo recruited 3,606 females from UMMC and SJMC who were at the clinic for opportunistic breast cancer screening between 2011 and 2016 [cohort described in [15]. MyMammo provided unmatched controls for MyBrCa, with 3,584 aged between 30 and 75 years at enrollment (**Additional file 4** – **Figure SM2B**).

Singapore Chinese Health Study (SCHS)

This prospective cohort recruited 63,257 Singaporean participants of Chinese ethnicity (mainly Southern Han Chinese of Hokkien and Cantonese dialect groups in Singapore) between 1993 and 1998 (https://sph.nus.edu.sg/research/cohort-schs/). Approximately 56% (n=35,298) were females aged between 45 and 74 years at enrollment. Genotyping information generated using the Illumina Global Screening Array was available for 11,280 women [17]; additional autosomal variants were imputed with IMPUTE v2 using the cosmopolitan 1000 Genomes reference panel (Phase 3). Linkage with the Singapore Cancer Registry was performed. A total of 418 breast cancer cases were diagnosed before 31 December 2015. SCHS excluded participants with a history of any cancer at enrollment, resulting in 10,213 available for this analysis (**Additional file 4** – **Figure SM3**). Protein truncating variants (PTV) were not studied in this cohort.

DNA isolation and genotyping

For SGBCC, MEC, MyBrCa, and MyMammo, DNA isolation was performed according to manufacturer's instructions for buffy coat samples for blood samples (FlexiGene DNA kit, Qiagen, or Promega's Maxwell 16 Blood DNA Purification Kit). DNA isolation for saliva samples was performed using Oragene and prepIT•L2P (DNA Genotek). DNA from peripheral blood in SCHS samples were extracted using QIAamp DNA Blood kits (Qiagen, Valencia, CA). Genotyping was performed on the Illumina OncoArray-500K Beadchip, as per previously described [18].

Polygenic risk score (PRS)

PRS is estimated as the weighted sum of effect alleles in 313 single nucleotide polymorphisms (SNPs) found to be associated with breast cancer; using plink (version 3) with the *scoresum* option (full details in **Supplement Methods** [19].

$$PRS = \beta_1 x_1 + \beta_2 x_2 + \cdots + \beta_k x_k + \cdots + \beta_{313} x_{313} ,$$

where x_k is the dosage of risk allele (0-2) for SNP k, β_k is the corresponding weight. The weights of the 313 SNPs for overall breast cancer risk were obtained from are of the overall breast cancer risk published by Mavaddat *et al*. (**Additional file 4** – **Table SM1**) [11]. Each individual's PRS was standardized to the mean and standard deviation of the PRS in controls by ethnicity (Chinese, Malay or Indian in the combined dataset of MEC and MyMammo). PRS of individuals of unknown ethnicity was standardized to the mean and standard deviation of all non-breast cancer controls in MEC and MyMammo (n=7,704). Effect allele frequencies by study are presented in **Additional file 4** – **Table SM1 (MEC) and Table SM2 (MyMammo)**.

Five-year absolute risk

Five-year absolute risk at the age of breast cancer diagnosis, for both PRS and the Gail model relative risk was estimated for breast cancer patients aged between 30 and 75 from SGBCC and MyBrCa. The absolute risk was based on ethnic-specific or overall breast cancer incidence rates (period of 2013 to 2017) for Singapore Citizens, and mortality rates (year 2016) in Singapore (**Additional file 3**) [20]. Both incidence and mortality rates were recorded in five-year intervals. The five-year absolute risk based on PRS was estimated using an iterative method detailed by Mavaddat *et al*. [13]. In brief, an individual's PRS percentile can be obtained from the standardized PRS using *pnorm* in R. The theoretical odds ratio of this percentile as compared to the 40-60 percentile (the closest in risk to the general population) can be estimated and subsequently the corresponding five-year absolute risk can be calculated [21]. The mean and standard deviations of PRS for SGBCC, MyBrCa, MEC and MyMammo are presented in **Additional file 4** – **Table SM3**. The five-year absolute breast cancer risk by PRS percentiles is illustrated in **Additional file 4** – **Figure SM4**.

The five-year absolute risk predicted by the Gail model was estimated using the methodology in the *BCRA* package in R [22]. Relative risk estimates for the risk factors estimated from the Asian American population were applied to Singapore and Malaysia. We applied the incidence and mortality rates of Singapore, to (1) account for differences in incidence and mortality rates between Asian Americans and the Asians from Singapore and Malaysia, and (2) to be consistent with the incidence and mortality rates used to estimate the absolute risk by PRS. To do this, the source code of the *BCRA* package was modified to reflect the breast cancer incidence rates of Chinese, Malays, Indians and all Singapore citizens, as well as the mortality rates of all Singapore citizens (**Additional file 3**). The five-year absolute risk (based on Gail model) of developing breast cancer for women at different ages is illustrated in **Additional file 4** – **Figure SM5**. The corresponding relative risk values are provided in **Additional file 4** – **Table SM4**.

Edited codes of BCRA package from R

The following code shows the edits done to the BCRA package from R. It requires:

1. The *BCRA* library [22]
2. The incidence and mortality rates of Singapore, provided as a separate file (**Additional file 3**)

Breast cancer incidence rates in the period 2013-2017, from the Singapore Cancer Registry, and age-specific mortality rates in 2016, from the Department of Statistics (Singapore) were used in the estimation of absolute risk [20, 23]. Incidence rates are ethnicity specific while mortality rates are based on all females.

Modified lines and regions are indicated by “EDITED”

```
lambda <- read.csv("Additional file 3.csv")

library(BCRA)

recode.check <- function (data, Raw_Ind = 1){
  Error_Ind <- rep(0, dim(data)[1])

  data$T1 <- as.numeric(as.character(data$T1)) ## EDITED
  data$T2 <- as.numeric(as.character(data$T2)) ## EDITED

  set_T1_missing <- data$T1
  set_T2_missing <- data$T2

  set_T1_missing[which((data$T1 < 20 | data$T1 >= 90) | data$T1 >=
```

```
                    data$T2)] <- NA
set_T2_missing[which(data$T2 > 90 | data$T1 >= data$T2)] <- NA
Error_Ind[is.na(set_T1_missing)] <- 1
Error_Ind[is.na(set_T2_missing)] <- 1
if (Raw_Ind == 1) {
  NB_Cat <- rep(-1, dim(data)[1])
  NB_Cat[which((data$N_Biop == 0 | data$N_Biop == 99) &
               data$HypPlas != 99)] <- "A"
  Error_Ind[which(NB_Cat == "A")] <- 1
  NB_Cat[which((data$N_Biop > 0 & data$N_Biop < 99) & (data$HypPlas !=
                                          0 & data$HypPlas != 1 & data$HypPlas != 99))] <- "B"
  Error_Ind[which(NB_Cat == "B")] <- 1
  NB_Cat[which(NB_Cat == -1 & (data$N_Biop == 0 | data$N_Biop ==
                              99))] <- 0
  NB_Cat[which(NB_Cat == -1 & data$N_Biop == 1)] <- 1
  NB_Cat[which(NB_Cat == -1 & (data$N_Biop >= 2 | data$N_Biop !=
                              99))] <- 2
  NB_Cat[which(NB_Cat == -1)] <- NA
  AM_Cat <- rep(NA, dim(data)[1])
  AM_Cat[which((data$AgeMen >= 14 & data$AgeMen <= data$T1) |
               data$AgeMen == 99)] <- 0
  AM_Cat[which(data$AgeMen >= 12 & data$AgeMen < 14)] <- 1
  AM_Cat[which(data$AgeMen > 0 & data$AgeMen < 12)] <- 2
  AM_Cat[which(data$AgeMen > data$T1 & data$AgeMen != 99)] <- NA
  AM_Cat[which(data$Race == 2 & AM_Cat == 2)] <- 1
  AF_Cat <- rep(NA, dim(data)[1])
  AF_Cat[which(data$Age1st < 20 | data$Age1st == 99)] <- 0
  AF_Cat[which(data$Age1st >= 20 & data$Age1st < 25)] <- 1
  AF_Cat[which((data$Age1st >= 25 & data$Age1st < 30) |
               data$Age1st == 98)] <- 2
  AF_Cat[which(data$Age1st >= 30 & data$Age1st < 98)] <- 3
  AF_Cat[which(data$Age1st < data$AgeMen & data$AgeMen !=
               99)] <- NA
  AF_Cat[which(data$Age1st > data$T1 & data$Age1st < 98)] <- NA
  AF_Cat[which(data$Race == 2)] <- 0
  NR_Cat <- rep(NA, dim(data)[1])
  NR_Cat[which(data$N_Rels == 0 | data$N_Rels == 99)] <- 0
  NR_Cat[which(data$N_Rels == 1)] <- 1
  NR_Cat[which(data$N_Rels >= 2 & data$N_Rels < 99)] <- 2
  NR_Cat[which((data$Race >= 6 & data$Race <= 11) & NR_Cat ==
               2)] <- 1
}
if (Raw_Ind == 0) {
  NB_Cat <- data$N_Biop
  AM_Cat <- data$AgeMen
  AF_Cat <- data$Age1st
  NR_Cat <- data$N_Rels
}
R_Hyp <- rep(NA, dim(data)[1])
R_Hyp[which(NB_Cat == 0)] <- 1
R_Hyp[which((NB_Cat != "A" & NB_Cat > 0) & data$HypPlas ==
             0)] <- 0.93
R_Hyp[which((NB_Cat != "A" & NB_Cat > 0) & data$HypPlas ==
             1)] <- 1.82
R_Hyp[which((NB_Cat != "A" & NB_Cat > 0) & data$HypPlas ==
             99)] <- 1
```

```
  set_HyperP_missing <- data$HypPlas
  set_R_Hyp_missing <- R_Hyp
  set_HyperP_missing[which(NB_Cat == "A")] <- "A"
  set_R_Hyp_missing[which(NB_Cat == "A")] <- "A"
  set_HyperP_missing[which(NB_Cat == "B")] <- "B"
  set_R_Hyp_missing[which(NB_Cat == "B")] <- "B"
  set_Race_missing <- data$Race
  Race_range <- seq(1, 15) ## EDITED
  set_Race_missing[-which(data$Race %in% Race_range)] <- "U"
  Error_Ind[which(is.na(NB_Cat) | is.na(AM_Cat) | is.na(AF_Cat) |
                  is.na(NR_Cat) | set_Race_missing == "U")] <- 1
  AF_Cat[which(data$Race == 2)] <- 0
  AM_Cat[which(data$Race == 2 & AM_Cat == 2)] <- 1
  NB_Cat[which((data$Race %in% c(3, 5)) & (data$N_Biop %in%
                                           c(0, 99)))] <- 0
  NB_Cat[which((data$Race %in% c(3, 5)) & NB_Cat == 2)] <- 1
  AM_Cat[which(data$Race == 3)] <- 0
  AF_Cat[which((data$Race %in% c(3, 5)) & (data$Age1st != 98) &
               AF_Cat == 2)] <- 1
  AF_Cat[which((data$Race %in% c(3, 5)) & AF_Cat == 3)] <- 2
  NR_Cat[which((data$Race %in% c(3, 5)) & NR_Cat == 2)] <- 1
  NR_Cat[which((data$Race >= 6 & data$Race <= 11) & NR_Cat ==
               2)] <- 1
  CharRace <- rep(NA, dim(data)[1])
  CharRace[which(data$Race == 1)] <- "Wh"
  CharRace[which(data$Race == 2)] <- "AA"
  CharRace[which(data$Race == 3)] <- "HU"
  CharRace[which(data$Race == 4)] <- "NA"
  CharRace[which(data$Race == 5)] <- "HF"
  CharRace[which(data$Race == 6)] <- "Ch"
  CharRace[which(data$Race == 7)] <- "Ja"
  CharRace[which(data$Race == 8)] <- "Fi"
  CharRace[which(data$Race == 9)] <- "Hw"
  CharRace[which(data$Race == 10)] <- "oP"
  CharRace[which(data$Race == 11)] <- "oA"
  CharRace[which(data$Race == 12)] <- "SG"  ## EDITED
  CharRace[which(data$Race == 13)] <- "CN"  ## EDITED
  CharRace[which(data$Race == 14)] <- "MY"  ## EDITED
  CharRace[which(data$Race == 15)] <- "IN"  ## EDITED
  CharRace[which(is.na(CharRace))] <- "??"
  recode_check <- cbind(Error_Ind, set_T1_missing, set_T2_missing,
                        NB_Cat, AM_Cat, AF_Cat, NR_Cat, R_Hyp, set_HyperP_missing,
                        set_R_Hyp_missing, set_Race_missing, CharRace)
  recode_check <- data.frame(recode_check, row.names = NULL)
  return(recode_check)
}

relative.risk <- function (data, Raw_Ind = 1){
  White_Beta <- c(0.5292641686, 0.0940103059, 0.2186262218,
                  0.9583027845, -0.288042483, -0.1908113865)
  Black_Beta <- c(0.1822121131, 0.2672530336, 0, 0.4757242578,
                  -0.1119411682, 0)
  Hspnc_Beta <- c(0.0970783641, 0, 0.2318368334, 0.166685441,
                  0, 0)
  FHspnc_Beta <- c(0.4798624017, 0.2593922322, 0.4669246218,
                   0.9076679727, 0, 0)
```

```
  Other_Beta <- c(0.5292641686, 0.0940103059, 0.2186262218,
                0.9583027845, -0.288042483, -0.1908113865)
  Asian_Beta <- c(0.55263612260619, 0.07499257592975, 0.27638268294593,
                0.79185633720481, 0, 0)
  Wrk_Beta_all <- rbind(White_Beta, Black_Beta, Hspnc_Beta,
                      Other_Beta, FHspnc_Beta, Asian_Beta, Asian_Beta, Asian_Beta,
                      Asian_Beta, Asian_Beta, Asian_Beta,
                      Asian_Beta, Asian_Beta, Asian_Beta, Asian_Beta) ## EDITED
  LP1 <- rep(NA, dim(data)[1])
  LP2 <- rep(NA, dim(data)[1])
  check_cov <- recode.check(data, Raw_Ind)
  NB_Cat <- check_cov$NB_Cat
  NB_Cat[which(NB_Cat == "A" | NB_Cat == "B")] <- NA
  NB_Cat <- as.numeric(as.character(NB_Cat))
  AM_Cat <- as.numeric(as.character(check_cov$AM_Cat))
  AF_Cat <- as.numeric(as.character(check_cov$AF_Cat))
  NR_Cat <- as.numeric(as.character(check_cov$NR_Cat))
  R_Hyp <- as.numeric(as.character(check_cov$R_Hyp))
  CharRace <- check_cov$CharRace
  PatternNumber <- rep(NA, dim(data)[1])
  PNID <- which(NB_Cat != "A" & NB_Cat != "B" &
                !is.na(AM_Cat) & !is.na(AF_Cat) & !is.na(NR_Cat))
  PatternNumber[PNID] <- NB_Cat[PNID] * 36 + AM_Cat[PNID] *
    12 + AF_Cat[PNID] * 3 + NR_Cat[PNID] * 1 + 1
  for (i in PNID) {
    if (CharRace[i] != "??") {
      Beta <- Wrk_Beta_all[data$Race[i], ]
      LP1[i] <- NB_Cat[i] * Beta[1] + AM_Cat[i] * Beta[2] +
        AF_Cat[i] * Beta[3] + NR_Cat[i] * Beta[4] + AF_Cat[i] *
        NR_Cat[i] * Beta[6] + log(R_Hyp[i])
      LP2[i] <- LP1[i] + NB_Cat[i] * Beta[5]
    }
  }
  RR_Star1 <- exp(LP1)
  RR_Star2 <- exp(LP2)
  RR_Star <- cbind(RR_Star1, RR_Star2, PatternNumber)
  RR_Star <- data.frame(RR_Star, row.names = NULL)
  return(RR_Star)
}


absolute.risk <- function (data, Raw_Ind = 1, Avg_White = 0){
  White_lambda1 <- c(1e-05, 7.6e-05, 0.000266, 0.000661, 0.001265,
                   0.001866, 0.002211, 0.002721, 0.003348, 0.003923, 0.004178,
                   0.004439, 0.004421, 0.004109)
  White_lambda1Avg <- c(1.22e-05, 7.41e-05, 0.0002297, 0.0005649,
                      0.0011645, 0.0019525, 0.0026154, 0.0030279, 0.0036757,
                      0.0042029, 0.0047308, 0.0049425, 0.0047976, 0.0040106)
  White_nlambda1 <- c(1.20469e-05, 7.46893e-05, 0.0002437767,
                    0.0005878291, 0.0012069622, 0.0019762053, 0.0026200977,
                    0.0033401788, 0.0039743676, 0.0044875763, 0.0048945499,
                    0.0051610641, 0.0048268456, 0.0040407389)
  Black_lambda1 <- c(2.696e-05, 0.00011295, 0.00031094, 0.00067639,
                   0.00119444, 0.00187394, 0.00241504, 0.00291112, 0.00310127,
                   0.0036656, 0.00393132, 0.00408951, 0.00396793, 0.00363712)
  Hspnc_lambda1 <- c(1.66e-05, 7.41e-05, 0.000274, 0.0006099,
```

```
          0.0012225, 0.0019027, 0.0023142, 0.0028357, 0.0031144,
          0.0030794, 0.0033344, 0.0035082, 0.0025308, 0.0020414)
Other_lambda1 <- c(1e-05, 7.6e-05, 0.000266, 0.000661, 0.001265,
          0.001866, 0.002211, 0.002721, 0.003348, 0.003923, 0.004178,
          0.004439, 0.004421, 0.004109)
FHspnc_lambda1 <- c(1.02e-05, 5.31e-05, 0.0001578, 0.0003602,
           0.0007617, 0.0011599, 0.0014111, 0.0017245, 0.0020619,
           0.0023603, 0.0025575, 0.0028227, 0.0028295, 0.0025868)
Chnes_lambda1 <- c(4.059636e-06, 4.5944465e-05, 0.000188279352,
          0.000492930493, 0.000913603501, 0.001471537353, 0.001421275482,
          0.001970946494, 0.001674745804, 0.001821581075, 0.001834477198,
          0.001919911972, 0.002233371071, 0.002247315779)
Japns_lambda1 <- c(1e-12, 9.9483924e-05, 0.000287041681,
          0.000545285759, 0.001152211095, 0.001859245108, 0.002606291272,
          0.003221751682, 0.004006961859, 0.003521715275, 0.003593038294,
          0.003589303081, 0.003538507159, 0.002051572909)
Filip_lambda1 <- c(7.500161e-06, 8.1073945e-05, 0.000227492565,
          0.000549786433, 0.001129400541, 0.001813873795, 0.002223665639,
          0.002680309266, 0.00289121923, 0.002534421279, 0.002457159409,
          0.00228661692, 0.001814802825, 0.00175087913)
Hawai_lambda1 <- c(4.5080582e-05, 9.8570724e-05, 0.00033997086,
          0.000852591429, 0.001668562761, 0.002552703284, 0.003321774046,
          0.005373001776, 0.005237808549, 0.005581732512, 0.005677419355,
          0.006513409962, 0.003889457523, 0.002949061662)
OtrPI_lambda1 <- c(1e-12, 7.1525212e-05, 0.000288799028,
          0.000602250698, 0.000755579402, 0.000766406354, 0.001893124938,
          0.002365580107, 0.00284393307, 0.002920921732, 0.002330395655,
          0.002036291235, 0.001482683983, 0.001012248203)
OtrAs_lambda1 <- c(1.2355409e-05, 5.9526456e-05, 0.000184320831,
          0.000454677273, 0.000791265338, 0.001048462801, 0.001372467817,
          0.001495473711, 0.001646746198, 0.001478363563, 0.001216010125,
          0.0010676637, 0.001376104012, 0.000661576644)

White_lambda2 <- c(0.000493, 0.000531, 0.000625, 0.000825,
          0.001307, 0.002181, 0.003655, 0.005852, 0.009439, 0.015028,
          0.023839, 0.038832, 0.066828, 0.144908)
White_lambda2Avg <- c(0.0004412, 0.0005254, 0.0006746, 0.0009092,
             0.0012534, 0.001957, 0.0032984, 0.0054622, 0.0091035,
             0.0141854, 0.0225935, 0.0361146, 0.0613626, 0.1420663)
White_nlambda2 <- c(0.0004000377, 0.0004280396, 0.0005656742,
           0.0008474486, 0.0012752947, 0.0018601059, 0.0028780622,
           0.0046903348, 0.0078835252, 0.0127434461, 0.0208586233,
           0.0335901145, 0.0575791439, 0.1377327125)
Black_lambda2 <- c(0.00074354, 0.00101698, 0.00145937, 0.00215933,
          0.00315077, 0.00448779, 0.00632281, 0.00963037, 0.01471818,
          0.02116304, 0.03266035, 0.04564087, 0.06835185, 0.13271262)
Hspnc_lambda2 <- c(0.0003561, 0.0004038, 0.0005281, 0.0008875,
          0.0013987, 0.0020769, 0.0030912, 0.004696, 0.007605,
          0.0120555, 0.0193805, 0.0288386, 0.0429634, 0.0740349)
Other_lambda2 <- c(0.000493, 0.000531, 0.000625, 0.000825,
          0.001307, 0.002181, 0.003655, 0.005852, 0.009439, 0.015028,
          0.023839, 0.038832, 0.066828, 0.144908)
FHspnc_lambda2 <- c(0.0003129, 0.0002908, 0.0003515, 0.0004943,
           0.0007807, 0.001284, 0.0020325, 0.0034533, 0.0058674,
           0.0096888, 0.0154429, 0.0254675, 0.0448037, 0.1125678)
Chnes_lambda2 <- c(0.000210649076, 0.000192644865, 0.000244435215,
```

```
                 0.000317895949, 0.000473261994, 0.00080027138, 0.001217480226,
                 0.002099836508, 0.003436889186, 0.006097405623, 0.010664526765,
                 0.020148678452, 0.03799079659, 0.098333900733)
Japns_lambda2 <- c(0.000173593803, 0.000295805882, 0.000228322534,
                 0.000363242389, 0.000590633044, 0.001086079485, 0.001859999966,
                 0.003216600974, 0.004719402141, 0.008535331402, 0.012433511681,
                 0.020230197885, 0.037725498348, 0.106149118663)
Filip_lambda2 <- c(0.000229120979, 0.000262988494, 0.00031484409,
                 0.000394471908, 0.00064762261, 0.001170202327, 0.001809380379,
                 0.002614170568, 0.004483330681, 0.007393665092, 0.012233059675,
                 0.021127058106, 0.037936954809, 0.085138518334)
Hawai_lambda2 <- c(0.000563507269, 0.000369640217, 0.001019912579,
                 0.001234013911, 0.002098344078, 0.002982934175, 0.005402445702,
                 0.009591474245, 0.016315472607, 0.020152229069, 0.02735483871,
                 0.050446998723, 0.072262026612, 0.145844504021)
OtrPI_lambda2 <- c(0.000465500812, 0.00060046692, 0.000851057138,
                 0.001478265376, 0.001931486788, 0.003866623959, 0.004924932309,
                 0.008177071806, 0.00863820289, 0.018974658371, 0.029257567105,
                 0.038408980974, 0.052869579345, 0.074745721133)
OtrAs_lambda2 <- c(0.000212632332, 0.000242170741, 0.000301552711,
                 0.000369053354, 0.000543002943, 0.000893862331, 0.001515172239,
                 0.002574669551, 0.004324370426, 0.007419621918, 0.01325176513,
                 0.02229142749, 0.041746550635, 0.087485802065)

#***EDITED (START)***#
SG_lambda1 <- lambda[,"BC_INCIDENCE"]/100000
SG_CN_lambda1 <- lambda[,"BC_INCIDENCE_CHINESE"] /100000
SG_MY_lambda1 <- lambda[,"BC_INCIDENCE_MALAY"] /100000
SG_IN_lambda1 <- lambda[,"BC_INCIDENCE_INDIAN"]/100000

SG_lambda2 <- lambda[,"DEATH_INCIDENCE"]/1000
#***EDITED (END)***#


White_1_AR <- c(0.5788413, 0.5788413)
Black_1_AR <- c(0.7294988, 0.74397137)
Hspnc_1_AR <- c(0.749294788397, 0.778215491668)
Other_1_AR <- c(0.5788413, 0.5788413)
FHspnc_1_AR <- c(0.428864989813, 0.450352338746)
Asian_1_AR <- c(0.47519806426735, 0.50316401683903)

Avg_lambda1 <- array(0, dim = c(14, 5))
Avg_lambda1[, 1:(dim(Avg_lambda1)[2])] <- White_lambda1Avg
Avg_lambda2 <- array(0, dim = c(14, 5))
Avg_lambda2[, 1:(dim(Avg_lambda2)[2])] <- White_lambda2Avg

Wrk_lambda1_all <- rbind(White_lambda1, Black_lambda1, Hspnc_lambda1,
                   Other_lambda1, FHspnc_lambda1, Chnes_lambda1, Japns_lambda1,
                   Filip_lambda1, Hawai_lambda1, OtrPI_lambda1, OtrAs_lambda1,
                   SG_lambda1,SG_CN_lambda1,SG_MY_lambda1,SG_IN_lambda1) ## EDITED
Wrk_lambda2_all <- rbind(White_lambda2, Black_lambda2, Hspnc_lambda2,
                   Other_lambda2, FHspnc_lambda2, Chnes_lambda2, Japns_lambda2,
                   Filip_lambda2, Hawai_lambda2, OtrPI_lambda2, OtrAs_lambda2,
                   SG_lambda2,SG_lambda2,SG_lambda2,SG_lambda2) ## EDITED
Wrk_1_AR_all <- rbind(White_1_AR, Black_1_AR, Hspnc_1_AR,
                  Other_1_AR, FHspnc_1_AR, Asian_1_AR, Asian_1_AR, Asian_1_AR,
```

```
                    Asian_1_AR, Asian_1_AR, Asian_1_AR,
                    Asian_1_AR,Asian_1_AR,Asian_1_AR,Asian_1_AR) ## EDITED

AbsRisk <- rep(NA, dim(data)[1])
RR_Star <- relative.risk(data, Raw_Ind)
check_cov <- recode.check(data, Raw_Ind)
Error_Ind <- check_cov$Error_Ind
IDwoERR <- which(Error_Ind == 0)
for (i in IDwoERR) {
  obs <- data[i, ]
  rrstar1 <- RR_Star$RR_Star1[i]
  rrstar2 <- RR_Star$RR_Star2[i]
  One_AR_RR <- rep(NA, 70)
  Strt_Intvl <- floor(obs$T1) - 20 + 1
  End_Intvl <- ceiling(obs$T2) - 20 + 0
  NumbrIntvl <- ceiling(obs$T2) - floor(obs$T1)
  RskWrk <- 0
  Cum_lambda <- 0
  lambda1.temp <- array(0, dim = c(14, 5))
  lambda2.temp <- array(0, dim = c(14, 5))

  #***EDITED (START)***#
  if(Avg_White==2){
    One_AR1 <- Wrk_1_AR_all[obs$Race, 1]
    One_AR2 <- Wrk_1_AR_all[obs$Race, 2]
    One_AR_RR1 <- One_AR1 * rrstar1
    One_AR_RR2 <- One_AR2 * rrstar2
    One_AR_RR[1:30] <- One_AR_RR1
    One_AR_RR[31:70] <- One_AR_RR2
    lambda1.temp[, 1:(dim(lambda1.temp)[2])] <- Wrk_lambda1_all[obs$Race,
    ]
    lambda2.temp[, 1:(dim(lambda2.temp)[2])] <- Wrk_lambda2_all[obs$Race,
    ]
    lambda1 <- c(t(lambda1.temp))
    lambda2 <- c(t(lambda2.temp))
  }
  #***EDITED (END)***#

  if (Avg_White == 0) {
    One_AR1 <- Wrk_1_AR_all[obs$Race, 1]
    One_AR2 <- Wrk_1_AR_all[obs$Race, 2]
    One_AR_RR1 <- One_AR1 * rrstar1
    One_AR_RR2 <- One_AR2 * rrstar2
    One_AR_RR[1:30] <- One_AR_RR1
    One_AR_RR[31:70] <- One_AR_RR2
    lambda1.temp[, 1:(dim(lambda1.temp)[2])] <- Wrk_lambda1_all[obs$Race,
    ]
    lambda2.temp[, 1:(dim(lambda2.temp)[2])] <- Wrk_lambda2_all[obs$Race,
    ]
    lambda1 <- c(t(lambda1.temp))
    lambda2 <- c(t(lambda2.temp))
  }
  if (Avg_White == 1) {
    One_AR_RR <- rep(1, 70)
    lambda1.temp[, 1:(dim(lambda1.temp)[2])] <- Wrk_lambda1_all[obs$Race,
    ]
```

```
    lambda2.temp[, 1:(dim(lambda2.temp)[2])] <- Wrk_lambda2_all[obs$Race,
    ]
    if (obs$Race == 1 | obs$Race == 4) {
      lambda1.temp <- Avg_lambda1
      lambda2.temp <- Avg_lambda2
    }
    lambda1 <- c(t(lambda1.temp))
    lambda2 <- c(t(lambda2.temp))
  }
  for (j in 1:NumbrIntvl) {
    j_intvl <- Strt_Intvl + j - 1
    if (NumbrIntvl > 1 & j > 1 & j < NumbrIntvl) {
      IntgrlLngth <- 1
    }
    if (NumbrIntvl > 1 & j == 1) {
      IntgrlLngth <- 1 - (obs$T1 - floor(obs$T1))
    }
    if (NumbrIntvl > 1 & j == NumbrIntvl) {
      z1 <- ifelse((obs$T2 > floor(obs$T2)), 1, 0)
      z2 <- ifelse((obs$T2 == floor(obs$T2)), 1, 0)
      IntgrlLngth <- (obs$T2 - floor(obs$T2)) * z1 +
        z2
    }
    if (NumbrIntvl == 1) {
      IntgrlLngth <- obs$T2 - obs$T1
    }
    lambdaj <- lambda1[j_intvl] * One_AR_RR[j_intvl] +
      lambda2[j_intvl]
    PI_j <- ((One_AR_RR[j_intvl] * lambda1[j_intvl]/lambdaj) *
              exp(-Cum_lambda)) * (1 - exp(-lambdaj * IntgrlLngth))
    RskWrk <- RskWrk + PI_j
    Cum_lambda <- Cum_lambda + lambdaj * IntgrlLngth
  }
  AbsRisk[i] <- 100 * RskWrk
 }
 return(AbsRisk)
}
```
